# Au(III) Extraction from Water Samples Using Triazole‐Coated Novel Magnetic Adsorbents and Analysis by Inductively Coupled Plasma‐Optical Emission Spectroscopy

**DOI:** 10.1002/open.202500161

**Published:** 2025-05-24

**Authors:** Celal Caner, Miraç Salpat, Salma Tabassum, Nuray Canikoglu, Huseyin Altundag

**Affiliations:** ^1^ Department of Chemistry Faculty of Science Sakarya University 54187 Sakarya Turkey; ^2^ Biomedical, Magnetic and Semiconductor Materials Research Center (BIMAS‐RC) Sakarya University 54187 Sakarya Turkey; ^3^ School of Environmental Science and Engineering Shanghai Jiao Tong University Shanghai 200240 China; ^4^ Department of Metallurgical and Materials Engineering Engineering Faculty Sakarya University 54187 Sakarya Turkey

**Keywords:** adsorption, gold, inductively coupled plasma‐optical emission spectroscopy, magnetic nanoparticles, triazole

## Abstract

In the present investigation, the synthesis, characterization, and application of triazole‐coated novel magnetic nanoparticles (MNPs) are systematically carried out, focusing on their efficacy as adsorbents for extracting Au(III) ions. The synthesis process involves the sequential coating of magnetite nanoparticles with tetraethylorthosilicate (SiO_2_), 3‐chloropropyltriethoxysilane (CPTES), and 3,5‐diamino‐1,2,4‐triazole (DAT). The MNPs synthesized at each stage are analyzed using high‐resolution transmission electron microscopy (HRTEM), field emission scanning electron microscopy (FESEM), energy‐dispersive X‐ray analysis (EDX), X‐ray diffraction (XRD), Fourier transform infrared spectroscopy (FT‐IR), and thermogravimetric and differential thermal analysis (DT/TGA) to confirm the successful coating of the desired adsorbent. Fe_3_O_4_@SiO_2_@CPTES@DAT MNPs selectively recover Au(III) ions under optimum conditions of pH = 2, 10 mg adsorbent amount, and 20 min contact time, and quantification of Au(III) ions is carried out by inductively coupled plasma‐optical emission spectroscopy (ICP‐OES). The method's suitability to adsorption isotherm and kinetic models is examined and found to be more compatible with the Freundlich isotherm and pseudo‐second‐order kinetic model. Relative standard deviation, limit of detection, and limit of quantification are calculated as 2.51%, 0.019, and 0.065 μg L^−1^, respectively, as analytical performance parameters. Several water samples are tested for Au(III) concentration using the optimized method.

## Graphical abstract

The synthesized magnetic nanoparticles, designated as Fe_3_O_4_@SiO_2_@CPTES@DAT, demonstrate effective recovery of Au(III) ions under optimized conditions at a pH of 2, utilizing 10 mg of the adsorbent, with an interaction time of 20 min. This finely tuned methodology is successfully employed to analyze various water samples regarding the recovery of Au(III).

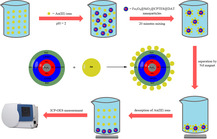

## 1 Introduction

Gold is highly valued for its economic and industrial significance, owing to its distinctive properties such as chemical resistance and electrical conductivity. Its widespread use in integrated circuits and electronic components further enhances its economic importance.^[^ ^1^ ^]^ As the demand for precious metals continues to escalate with industrial development, the availability of gold resources is increasingly insufficient, resulting in a continuous upward trajectory in its market price.^[^ ^2^ ^]^ Consequently, the extraction of gold from wastewater is vital for advancing resource utilization and promoting sustainable practices in material recovery.^[^ ^3^ ^]^ A range of methodologies, including solvent extraction,^[^ ^4^ ^]^ membrane separation,^[^ ^5^ ^]^ ion exchange,^[^ ^6^ ^]^ precipitation,^[^ ^7^ ^]^ and adsorption,^[^ ^8^ ^]^ have been established to recover Au(III) ions from wastewater efficiently. These techniques offer promising avenues for enhancing the recovery processes in environmental applications.^[^ ^9^ ^]^

Gold recovery from water samples poses significant challenges, mainly due to various contaminants such as iron, nickel, zinc, and manganese. These contaminants can impede the adsorption process, thereby reducing the efficiency of gold extraction.^[^ ^10^ ^]^ Other metals, high ionic strength, and competitive heavy metals can also hinder gold recovery.^[^ ^11^ ^]^ These challenges underscore the need for advanced methods and technologies to enhance gold recovery from water samples and promote environmental sustainability.^[^ ^12^ ^]^ Adsorption is the process by which ions, molecules, or atoms in a liquid or gaseous state are deposited on the surface of a solid or liquid to form a thin layer. This process can occur through physical forces (physical adsorption) or chemical bonds (chemical adsorption).^[^ ^13^ ^]^ Adsorption is widely preferred due to its efficiency, cost‐effectiveness, and ease of application compared to other separation techniques.^[^ ^14^ ^]^

Magnetic nanoparticles (MNPs) are increasingly utilized for gold adsorption because of their beneficial characteristics. Their high surface area enhances adsorption capacity, while their superparamagnetic properties enable easy separation from solutions using an external magnetic field, making the process more efficient and reusable.^[^ ^15^ ^]^ Additionally, MNPs can be functionalized for specific targets, such as gold, improving selectivity and adsorption efficiency.^[^ ^16^ ^]^ MNPs are versatile materials suitable for various environmental and industrial applications, including removing heavy metals and organic pollutants.^[^ ^17^, ^18^ ^]^ In gold adsorption, MNPs reduce operational costs and environmental impacts due to their easy separability and reusability, and they enhance the adsorption process by forming complexes with gold ions.^[^ ^19^ ^]^

MNPs, in their uncoated state, are prone to oxidation, tend to agglomerate and can degrade easily in highly acidic conditions.^[^ ^20^ ^]^ However, when they are coated with suitable materials, their resistance to various external environments improves, and they can interact more effectively with target metals, mainly when functional groups containing oxygen (O), nitrogen (N), or sulfur (S) are present.^[^ ^21^ ^]^ In this study, Fe_3_O_4_ nanoparticles were coated with tetraethylorthosilicate (SiO_2_), 3‐chloropropyltriethoxysilane (CPTES), and 3,5‐diamino‐1,2,4‐triazole (DAT), thereby enhancing their resilience against external factors and interacting with an outside shell of the nanoparticles, which contains plenty of N atoms.

The present study involves the synthesis and characterization of triazole‐coated MNPs and the recovery of Au(III) ions from water samples. Fe_3_O_4_ nanoparticles were coated with SiO_2_ to make them resistant to aggregation and oxidation, then coated with CPTES to functionalize them with chlorine atoms, and finally coated with DAT to make them suitable for the recovery of Au(III) ions. The reaction products of each step were characterized by high‐resolution transmission electron microscopy (HRTEM), field emission scanning electron microscopy (FESEM), energy‐dispersive X‐ray analysis (EDX), X‐ray diffraction (XRD), Fourier transform infrared spectroscopy (FT‐IR), and thermogravimetric and differential thermal analysis (TGA) to prove that the coating was successful. Fe_3_O_4_@SiO_2_@CPTES@DAT nanoparticles synthesized in the magnetic solid‐phase extraction method were used in Au(III) recovery, and parameters such as solution pH, adsorbent amount, and contact time were optimized. In addition, Au(III) ions were removed from the adsorbent surface using an appropriate desorption solution. Different adsorption isotherm and kinetic studies were performed, and the maximum adsorption capacity was calculated. Furthermore, the recovery percentages of Au(III) ions in the presence of different foreign ions at varying concentrations of solutions subjected to magnetic solid phase extraction were investigated. After calculating analytical performance metrics like relative standard deviation (RSD), limit of detection (LOD), and limit of quantification (LOQ), the technique was effectively used to recover Au(III) from various water samples.

## 2 Experimental Section

### 2.1 Instrumentation

The nanoparticles’ surface morphology and average size were analyzed using the FEI TALOS F200S TEM 200 kV (Thermo Fisher Scientific, USA) and Quanta 450 FEG FESEM (FEI, Japan), and their chemical composition was determined using the EDX coupled to FESEM (SEM‐JEOL‐JSM 6060LV). The crystal structure of the nanoparticles was studied using the DMAX‐2000 XRD (Rigaku D, Japan). The FT‐IR spectra of the nanoparticles were obtained using the Spectrum 2 FT‐IR spectrometer (Perkin Elmer, USA). TGA were carried out with the SDT Q600 (TA Instruments, Germany). The concentration of Au(III) was measured using the Spectro Arcos FHE‐16 inductively coupled plasma‐optical emission spectroscopy (ICP‐OES) (Spectro, Germany). pH values were determined using the Orion 2‐Star pH meter (Thermo Fisher Scientific, USA). The Milli‐Q Advantage A10 water purification system (Merck, Germany) provided ultrapure water to prepare all solutions. Dissolution was performed using the Bandelin Sonorex Super RK 225 ultrasonic bath (Bandelin, Germany). MNPs were separated using a neodymium–iron–boron (Nd–Fe–B) magnet (40 × 20 × 10 mm). All of the glassware was immersed in a 3 mol L^−1^ nitric acid solution and washed with distilled water before experimenting.

### 2.2 Materials and Reagents

Chemicals were purchased from Merck (Germany). Analytical purity chemicals were utilized in all experiments, and no additional purification was conducted—the synthesis of Fe_3_O_4_ employed iron (III) chloride hexahydrate and iron (II) chloride tetrahydrate. SiO_2_, CPTES, and DAT were used in the nanoparticle coating processes. The stock gold solution was diluted daily to prepare gold (III) standard solutions. pH adjustments of the solutions were carried out using 0.1 mol L^−1^ HCl or 0.1 mol L^−1^ NaOH. Ethanol, ammonia, dimethylformamide, and triethylamine were solvents in the nanoparticle coating processes.

### 2.3 Fe_3_O_4_@SiO_2_@CPTES@DAT Synthesis

The procedure involved dissolving 5.2 grams of FeCl_3_.6H_2_O and 2 grams of FeCl_2_.4H_2_O in 25 mL of 0.4 mol L^−1^ HCl acid in an ultrasonic bath. Simultaneously, 250 mL of 1.5 mol L^−1^ NaOH solution was prepared. The iron solution was then added dropwise over 30 min to the continuously stirred base solution under a nitrogen atmosphere at 70°C. After stirring for another half hour, the mixture was removed from the reaction medium with a magnet, cleaned until the pH was neutral, and dried at 60°C in an oven. After the reaction, 2.34 grams of product were obtained. The coating process began with dispersing two grams of the synthesized Fe_3_O_4_ nanoparticles in a 100 mL water/ethanol (1:4) mixture for 30 min. Subsequently, the mixture was stirred for 24 h at room temperature after adding 2 mL of TEOS solution and 3 mL of 25% concentrated ammonia. Following the reaction, 2.58 grams of product were produced by washing the nanoparticles with ethanol using magnetic decantation and then drying them in an oven set at 60°C for 24 h.

2 g of Fe_3_O_4_@SiO_2_ nanoparticles were dispersed in 100 mL of ethanol for 30 min. Dropwise additions of 2 mL of CPTES and 5 mL of pure ammonia were then performed. After that, the mixture was stirred for 24 h at room temperature. Subsequently, 50 mL of ethanol was used five times to wash the reaction mixture to remove unreacted products. Finally, 1.87 g of the product was obtained after being dried for 24 h at 60°C.

**Figure 1:**
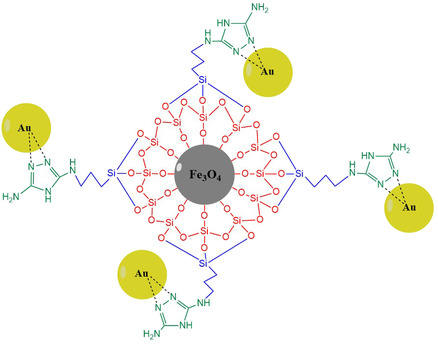
A schematic representation of Fe_3_O_4_@SiO_2_@CPTES@DAT nanoparticles’ adsorption of Au(III) ions.

In the final stage of the reaction, 1 mL of triethylamine was added after 0.495 g (5 mmol) of DAT had been dissolved in 50 mL of toluene. Following the addition of 1 g of Fe_3_O_4_@SiO_2_@CPTES, the reaction mixture was heated to 120 °C. The reaction was then refluxed in a nitrogen atmosphere for 24 h. Afterward, the synthesized nanoparticles were separated from the solution with a magnet, cleaned five times with 10 mL of DMF, and dried for 24 h at 60 °C in an oven. The reaction yielded 0.95 g of the product.^[^ ^22^ ^]^ **Figure** **1** demonstrates the chemical structure of Fe_3_O_4_@SiO_2_@CPTES@DAT nanoparticles and the Au(III) adsorption mechanism.

### 2.4 Extraction Procedure

Magnetic solid‐phase extraction was utilized for solutions containing a 1000 μg L^−1^ Au(III) solution. Using 0.1 mol L^−1^ HCl or 0.1 mol L^−1^ NaOH solutions, the pH of the solution was brought down to 2. Following the addition of 10 mg of Fe_3_O_4_@SiO_2_@CPTES@DAT, the mixture was stirred for 20 min at 150 rpm. For 20 min, the solution was agitated at 150 rpm, while 10 mg of Fe_3_O_4_@SiO_2_@CPTES@DAT was added. This allowed the Au(III) ions to be adsorbed onto the nanoparticles. The adsorbent was then separated using an Nd magnet, and the remaining solution was removed by decantation. The concentration of Au(III) in the separated solution was then assessed by ICP‐OES.^[^ ^23^ ^]^

## 3 Results and Discussion

### 3.1 Characterization

#### 3.1.1 HRTEM

**Figure 2:**
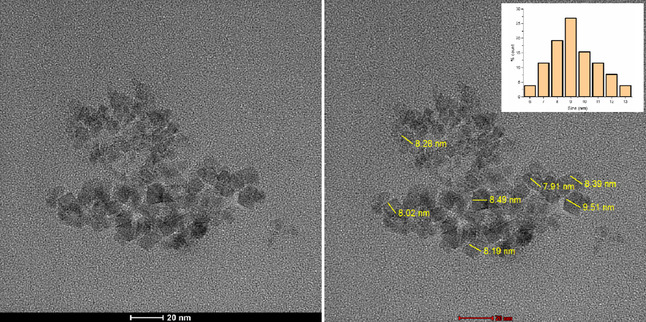
HRTEM image of Fe_3_O_4_@SiO_2_@CPTES@DAT nanoparticles.

HRTEM is an advanced imaging technique that enables direct observation of materials at or near the atomic scale.^[^ ^24^ ^]^ Correlating HRTEM images with other spectroscopic techniques, the exchange coupling behavior and surface effects in MNPs can be studied, making it possible to understand the relationship between structural properties and magnetic properties.^[^ ^25^ ^]^ The morphology and size of Fe_3_O_4_@SiO_2_@CPTES@DAT nanoparticles were evaluated using HRTEM. The HRTEM image in **Figure** **2** illustrates nanoparticles with a spherical core‐shell structure, with an average size of 9 nm. The image reveals varying electron densities, with darker regions indicating the presence of MNPs and lighter regions corresponding to the coating. For this analysis, 28 nanoparticles were randomly selected from the TEM images, and ImageJ software was used to measure the average thickness of the silica layer.

#### 3.1.2 FESEM

**Figure 3:**
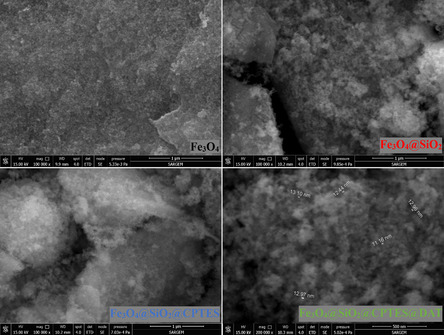
FESEM images of synthesized nanoparticles.

FESEM is an advanced imaging technique used to analyze surface structures. It involves directing a focused electron beam is directed toward the sample surface to capture detailed 3D images at the nanometer scale.^[^ ^26^ ^]^ With a magnification capability of over 100000 x, FESEM is well‐suited for observing fine details of MNPs, which are usually between 2 and 50 nm in size.^[^ ^27^ ^]^ In the study, FESEM images of Fe_3_O_4_, Fe_3_O_4_@SiO_2_, Fe_3_O_4_@SiO_2_@CPTES, and Fe_3_O_4_@SiO_2_@CPTES@DAT nanoparticles are illustrated in **Figure** **3**, with a focus on examining the surface interactions and structural properties of the nanoparticles. It was observed that Fe_3_O_4_ nanoparticles tended to aggregate in their bare state, but their surfaces became rough when coated with SiO_2_. The electron density in the images suggests that they were synthesized in a core‐shell structure.

#### 3.1.3 EDX

**Figure 4:**
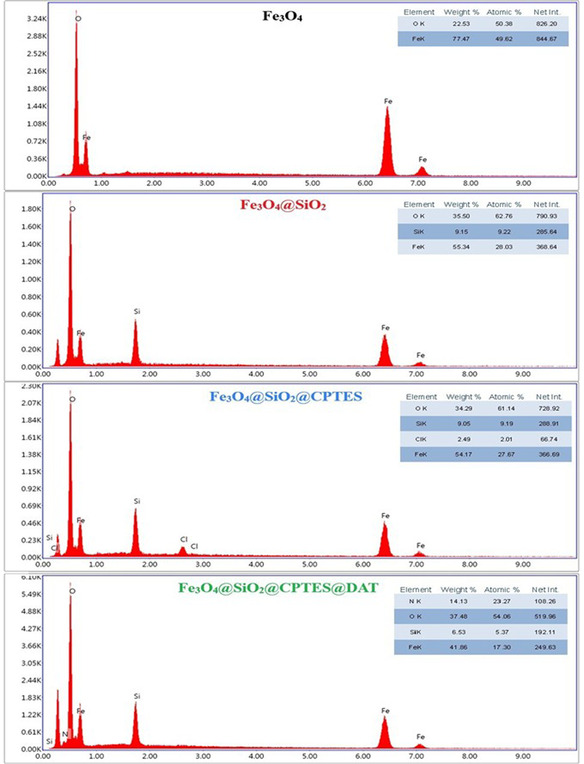
EDX spectra of synthesized nanoparticles.

For multielement analysis, EDX is a nondestructive analytical technique. It allows for determining the elemental composition of a sample by measuring the energy of characteristic X‐rays emitted after irradiation of the sample with X‐rays and requires no special preparation. EDX is user‐friendly, rapid, and cost‐effective, making it suitable for screening metals and other materials in various fields such as industry and forensics.^[^ ^28^ ^]^ Additionally, EDX provides valuable insights into the elemental composition of MNPs, aiding in understanding their properties and the success of advanced coatings. **Figure** **4** displays the EDX spectra of the synthesized nanoparticles. The spectrum of Fe_3_O_4_ nanoparticles reveals the presence of 77.47 wt% Fe and 22.53 wt% O atoms, while the spectrum of Fe_3_O_4_@SiO_2_ represents 55.34 wt% Fe, 35.5 wt% O, and 9.15 wt% Si atoms. The Si and intensified O peaks indicate the successful SiO_2_ coating process. Also, the spectrum of Fe_3_O_4_@SiO_2_@CPTES illustrates 54.17 wt% Fe, 34.29 wt% O, 9.05 wt% Si, and 2.49 wt% Cl atoms, the Cl peaks demonstrating the successful CPTES coating of the nanoparticle. In the Fe_3_O_4_@SiO_2_@CPTES@DAT spectrum, 41.86 wt% Fe, 37.48 wt% O, 6.53 wt% Si, and 14.13 wt% N atoms are present, with the distinct N peaks indicating the successful triazole coating process.

#### 3.1.4 XRD

**Figure 5:**
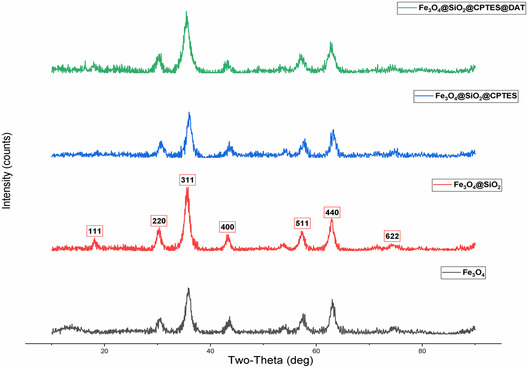
XRD spectra of synthesized nanoparticles.

XRD is a powerful analytical technique used to determine the crystal structure of materials. It delivers comprehensive details regarding the crystallographic structure, phase identification, and other structural properties of the material by analyzing the diffraction patterns obtained from directing X‐rays at the material.^[^ ^29^ ^]^ XRD can confirm the formation of the cubic phase, which is essential for magnetic properties, and measure crystallite sizes using the Scherrer equation.^[^ ^30^ ^]^ The XRD spectra of the nanoparticles synthesized in the study are shown in **Figure** **5**, 18.62, 30.38, 35.94, 43.85, 57.50, 62.99, and 75.87 peaks at 2θ angles corresponding to lattice planes (111), (220), (311), (400), (511), (440), and (622) respectively. The synthesized nanoparticle has JCPDS card number 01‐075‐0449, corresponding to the spinel face‐centered cubic structure of Fe_3_O_4_. The nanoparticle size calculated using Scherrer's equation is 12.18 nm, and the lattice constant of the crystal structure is 0.8385 nm. The outcomes validate that the Fe_3_O_4_@SiO_2_@CPTES@DAT nanoparticle is coated without degradation, consistent with the values reported in the literature.

#### 3.1.5 FT‐IR

**Figure 6:**
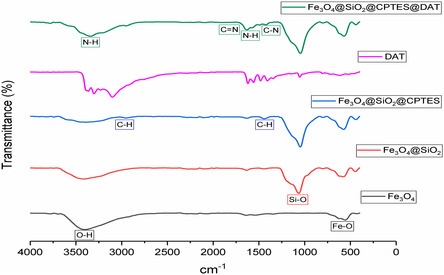
FT‐IR spectra of synthesized nanoparticles.

FT‐IR is a widely used analytical method for determining the molecular composition and structural properties of materials by measuring the vibrations of molecular bonds under infrared rays.^[^ ^31^ ^]^ It is commonly employed to verify the structural integrity of MNPs and the successful application of desired modifications.^[^ ^32^ ^]^ The synthesized nanoparticles’ FT‐IR spectra can be seen in **Figure** **6**, providing information about the functional groups contained in the nanoparticles. The Fe_3_O_4_ spectrum shows O‐H stretching vibrations at 3398 cm^−1^ and Fe–O stretching vibrations at 555–627 cm^−1^. The 1065–1152 cm^−1^ bands in the Fe_3_O_4_@SiO_2_ spectrum belong to Si–O–Si stretching vibrations. The CPTES molecule's binding to the structure is indicated by the 2957 and 1441 cm^−1^ bands in the Fe_3_O_4_@SiO_2_@CPTES spectrum, which correspond to C–H stretching and bending vibrations, respectively. N–H stretching vibrations are represented by the 3337 cm^−1^ band in the Fe_3_O_4_@SiO_2_@CPTES@DAT spectrum, C = N stretching by the 1627 and 1570 cm^−1^ bands, N–H bending by the 1570 cm^−1^ band, and C–N stretching vibrations by the 1418 cm^−1^ band. The presence of peaks similar to those of the DAT molecule confirms the successful synthesis and coating of the nanoparticle.

#### 3.1.6 TGA

TGA is an analytical technique that measures changes in the physical and chemical properties of materials as a function of temperature and/or time. It is mainly used to determine the properties of a material, such as degradation, oxidation, or loss of volatiles (e.g., moisture).^[^ ^33^ ^]^ TGA contributes significantly to comprehending the stability of the material and its behavior under different conditions by determining the organic/inorganic chemical composition of MNPs.^[^ ^34^ ^]^ DTA is a technique used to measure the temperature difference between a sample and a reference substance under controlled conditions.^[^ ^35^ ^]^

**Figure 7:**
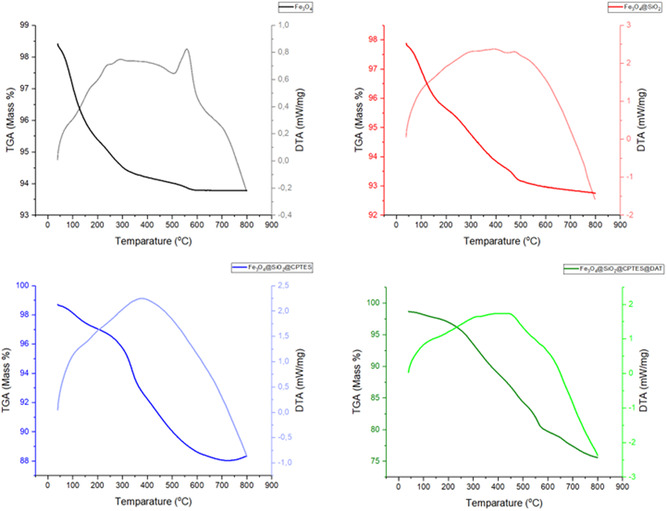
TGA and DTA spectra of synthesized nanoparticles.

**Figure** **7** shows the TGA and DTA spectra of the synthesized nanoparticles, which were heated at a rate of 10 °C per minute from ambient temperature to 800 °C. The nanoparticles’ mass decreased as the temperature rose during this operation. The TGA spectrum for Fe_3_O_4_ indicated a mass loss of 2.73% within the temperature range of 40–170 °C, attributed to water or solvent molecules that were physically adsorbed to the sample. In the DTA spectrum for Fe_3_O_4_, an endothermic peak was identified in the 510–600 °C range, indicating the conversion of magnetite (Fe_3_O_4_) to hematite (Fe_2_O_3_). For the Fe_3_O_4_@SiO_2_ TGA spectrum, a mass loss of 2.65% was observed between 170 and 490 °C, resulting from the decomposition of SiO_2_ molecules. In the case of the Fe_3_O_4_@SiO_2_@CPTES TGA spectrum, there was a mass loss of 7.77% between 250 and 580 °C, which differs from the Fe_3_O_4_@SiO_2_ spectrum and corresponds to the decomposition of CPTES molecules. Finally, in the Fe_3_O_4_@SiO_2_@CPTES@DAT TGA spectrum, a mass loss of 15.26% was recorded in the 260–570 °C range, linked to the decomposition of DAT molecules.

### 3.2 Optimization Studies

#### 3.2.1 pH

**Figure 8:**
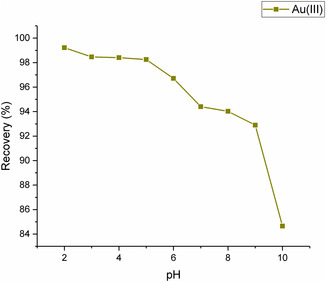
The impact of pH on Au(III) ions adsorption (adsorbent amount 10 mg, contact time 20 min, Au(III) concentration of 1000 μg L^−1^).

Depending on the type of MNPs and the metal involved, pH significantly impacts the recovery of heavy metals by MNPs. Due to its impact on the surface charge of both the analyte and the adsorbent, pH also influences the adsorption mechanisms for certain metals. The complexation mechanism becomes more significant at higher pH values, whereas cation exchange is essential at lower pH values.^[^ ^36^ ^]^ MNPs can be reused with minimal loss in adsorption capacity, which is typically pH‐dependent. The study investigated samples with pH values ranging from 2 to 10, and it was discovered that pH 2 resulted in the optimal recovery of Au(III) ions. As shown in **Figure** **8**, Au(III) ions exhibited high recovery rates at pH 2, while the recovery decreased with increasing pH. Rahmanyanti et al. synthesized MNPs modified with gallic acid and adsorbed [AuCl_4_]^−^ ions.^[^ ^37^ ^]^ The study's pH range was 2–7, with pH 3 yielding the highest recovery. They explained that the nanoparticles would become more negatively charged due to the ionization of ‐OH groups on gallic acid above pH 3, leading to decreased adsorption.

#### 3.2.2 Amount of Adsorbent

**Figure 9:**
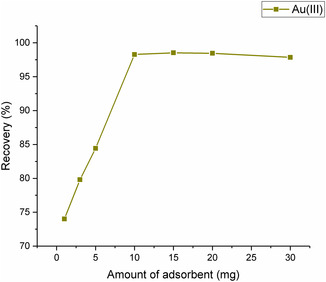
Amount of adsorbent effect on Au(III) ions adsorption (pH 2, contact time 20 min, Au(III) concentration of 1000 μg L^−1^).

The effectiveness of gold adsorption by MNPs is greatly influenced by the amount of adsorbent utilized. Typically, higher amounts of adsorbent can enhance the removal efficiency of heavy metals. However, an optimal quantity of adsorbent exists for each metal and nanoparticle. Excessive adsorbent amounts can lead to reduced efficiency due to a decrease in surface area caused by nanoparticle aggregation.^[^ ^14^ ^]^ In the study, Fe_3_O_4_@SiO_2_@CPTES@DAT was introduced to a solution containing Au(III) ions in quantities ranging from 1 to 30 mg, and the adsorption occurred and recovery rates were calculated. **Figure** **9** illustrates that an adsorbent amount of 10 mg and above resulted in a recovery rate of 95% or higher, and this amount was subsequently utilized in further studies. Recently, Poudel et al. developed an adsorbent by polymerizing pyrrole‐1‐carbodithioic acid on silica‐coated Fe_3_O_4_ nanoparticles and found that the maximum sorption and recovery of Ru, Rh, Ir, Pd, Pt, and Au occurred in the presence of 15 mg of adsorbent.^[^ ^38^ ^]^

#### 3.2.3 Contact Time

**Figure 10:**
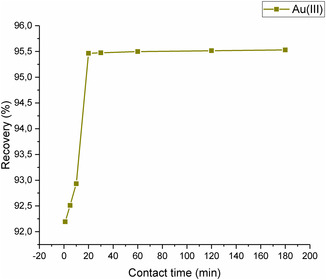
Impact of contact time on Au(III) ion adsorption (pH 2, adsorbent amount 10 mg, Au(III) concentration of 1000 μg L^−1^).

In gold recovery using MNPs, contact time is a factor that significantly affects adsorption efficiency. Studies show that as the contact time increases, the adsorption efficiency increases up to a certain point.^[^ ^39^ ^]^ In this investigation, the recovery of Au(III) ions at different contact times between 1 and 180 min was investigated and depicted in **Figure** **10**. It was observed that even at very short times, high extraction efficiency was reached. Therefore, for further studies, 20 min was selected as the optimum contact time. Khoshhesab et al. synthesized nickel ferrite (NiFe_2_O_4_) MNPs and realized the recovery of Au(III) ions from water samples by applying a contact time of 30 min.^[^ ^40^ ^]^

#### 3.2.4 Desorption

Desorption is essential for the recovery and reuse of MNPs in the removal of heavy metals. The selection of the desorption agent is a critical factor in this process. Generally, desorption occurs through changes in pH by breaking the bonds that exist between the metal ions and the functional groups on the MNPs, often facilitated by specific chemical agents.^[^ ^41^ ^]^ An effective desorption process not only enhances the reusability of MNPs but also sustains their adsorption capacity, thereby improving overall process efficiency. In this study, various concentrations of acidic and basic desorption agents containing thiourea were employed. Specifically, Au(III) ions were quantitatively extracted from the Fe_3_O_4_@SiO_2_@CPTES@DAT nanoparticles using a 1 M HCl solution combined with 0.1 M thiourea, as seen in **Table** **1**. Moreover, Lin et al. synthesized thiourea‐modified MNPs that successfully adsorbed Au(III) ions in an appropriate medium and used a 2 M HCl solution containing 0.7 M thiourea for desorption.^[^ ^42^ ^]^

**Table 1:** Au(III) recoveries at various desorption agent concentrations (pH 2, adsorbent amount 10 mg, contact time 20 min, Au(III) concentration of 1000 μg L^−1^).

Concentration [mol L^−1^]	HCl (0.1 M Thiourea)	NaOH
0.25	91.25	2.29
0.5	93.69	4.85
1	97.94	8.16
1.5	98.81	9.38

#### 3.2.5 Foreign Ion Effect

In studies examining foreign ions, interfering ions were introduced into aqueous solutions containing 1 mg L^−1^ of Au(III) ions at various concentrations to evaluate their impact on recovery. The tolerance values for these ions and the corresponding percentages of Au(III) recovery are detailed in **Table** **2**. The findings reveal that substantial recovery rates of Au(III) can be achieved, even when foreign ions are present in high concentrations, suggesting the feasibility of conducting the study in diverse matrix environments. Zhao et al. developed MNPs modified with poly(1‐vinylimidazole) for the selective adsorption of Au(III) ions.^[^ ^43^ ^]^ Their research indicates that Au(III) ions can be effectively recovered from the solution with high selectivity, despite the presence of additional ions like Cd(II), Cu(II), Hg(II), Pd(II), and Pt(IV).

**Table 2:** Foreign ion effect on Au(III) adsorption (pH 2, adsorbent amount 10 mg, contact time 20 min, Au(III) concentration of 1000 μg L^−1^).

Foreign ion	Concentration [μg mL^−1^]	Recovery of Au(III) [%]
Na^+^	1000	95.7
K^+^	1000	97.4
Ca^2+^	100	96.1
Mg^2+^	200	96.3
Fe^3+^	50	95.2
Cu^2+^	50	96.8
Ni^2+^	25	95.2
Cl^−^	250	97.8
NH_4_^+^	250	96.5
NO_3_^−^	1000	97.4
SO_4_^2−^	1000	95.4
CH_3_COO^−^	250	97.1

#### 3.2.6 Adsorption Isotherms

Adsorption isotherms characterize the interactions between adsorbates and the adsorbent surface, playing a vital role in these processes. The Freundlich, Temkin, and Langmuir isotherms are the three main isotherms that rely on different assumptions regarding the surface properties. The Langmuir isotherm posits that adsorption takes place as a monolayer on a homogeneous surface, and the quantity of adsorption can be represented by the following equation

(1) $$\frac{c_{e}}{q_{e}}=\frac{1}{K_{L}.q_{\text{max}}}+\frac{c_{e}}{q_{\text{max}}}$$

where *q*_max_ denotes the maximum adsorption capacity, while *K*_L_ reflects the Langmuir constant.^[^ ^44^ ^]^ Conversely, multilayer adsorption processes on heterogeneous surfaces are described by the Freundlich isotherm. This model operates under the premise that adsorption transpires on surfaces exhibiting variable energy levels. The Freundlich isotherm is mathematically represented by a specific equation

(2) $$\log q_{e}=\log K_{F}+\frac{1}{n}\text{log}C_{e}$$

where *K*_F_ denotes the adsorption capacity and 1/*n* represents the degree of surface heterogeneity.^[^ ^45^ ^]^ During the adsorption process, the interactions between the adsorbent and the adsorbate are taken into consideration using the Temkin isotherm. It is based on the assumption that the adsorption energy decreases linearly with surface occupancy, and the following formula represents it

(3) $$\text{qe}=\frac{\text{RT}}{b}\ln K_{T}+\frac{\text{RT}}{b}\text{ln}c_{e}$$

where the constants *b* and *K*_T_ are indicative of the adsorption energy. Hence, the Langmuir isotherm is appropriate for modeling adsorption on homogeneous surfaces, while the Freundlich isotherm is more suitable for multilayer adsorption occurring on heterogeneous surfaces.^[^ ^46^ ^]^ Additionally, the Temkin isotherm accounts for interactions between adsorbates, making it relevant for surfaces where such interactions are significant.

**Figure 11:**
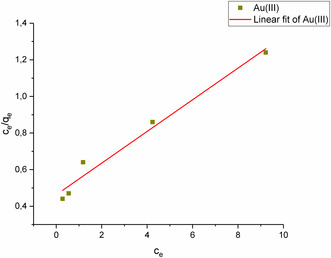
Langmuir isotherm graph of Au(III) ions (pH 2, adsorbent amount 10 mg, contact time 20 min, Au(III) concentration of 1000–50 000 μg L^−1^, temperature 298 K).

**Figure 12:**
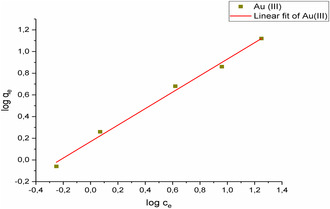
Freundlich isotherm graph of Au(III) ions (pH 2, adsorbent amount 10 mg, contact time 20 min, Au(III) concentration of 1000–50 000 μg L^−1^, temperature 298 K).

In this study, adsorption was performed on Au(III) solutions with concentrations varying from 1 to 50 mg L^−1^, and isotherm modeling was conducted to explore equilibrium adsorption. **Figure** **11**, **12**, **13** depict the linear Langmuir, Freundlich, and Temkin isotherm models for Au(III) ions, respectively. While all three models demonstrated high correlation coefficient values, the best fit to the data was found to be the Freundlich isotherm (R^2^ > 0.99). This indicates that Au(III) ions are adsorbed in multiple layers onto the Fe_3_O_4_@SiO_2_@CPTES@DAT adsorbent surface. **Table** **3** provides the calculated isotherm parameters, equations, and correlation coefficients for each isotherm model.

**Figure 13:**
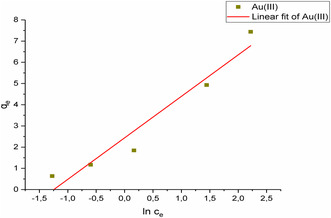
Temkin isotherm graph of Au(III) ions (pH 2, adsorbent amount 10 mg, Au(III) concentration of 1000–50 000 μg L^−1^, temperature 298 K).

**Table 3:** Langmuir, Freundlich, and Temkin isotherm parameters for Au(III) adsorption (pH 2, adsorbent amount 10 mg, Au(III) concentration of 1000–50 000 μg L^−1^, temperature 298 K).

Langmuir isotherm	q_m_ [mg g^−1^]	K_L_ [L mg^−1^]	R^2^	Equation
	11.6	0.187	0.975	*y* = 0.0865*x* + 0.462
Freundlich isotherm	K_f_ (L mg^−1^)	1/n	R^2^	Equation
	1.48	0.76	0.993	*y* = 0.760*x* + 0.169
Temkin isotherm	b	K_T_ (L mol^−1^)	R^2^	Equation
	127	3.47	0.944	*y* = 1.95*x* + 2.43

#### 3.2.7 Adsorption Kinetics

Adsorption kinetics refers to the rate at which a dissolved substance adheres to the surface of an adsorbent. Two of the most commonly used models to describe this process are the pseudo‐first‐order and pseudo‐second‐order kinetic models. In the pseudo‐first‐order kinetic model, the rate of filling adsorption sites is assumed to be proportional to the number of unfilled sites. This model is typically represented by the Lagergren equation.^[^ ^47^ ^]^ Although it is valued for simplicity, this model may not adequately account for systems with more complex interactions.

Conversely, the pseudo‐second‐order kinetic model posits that the adsorption rate is proportional to the square of the number of unoccupied sites. This model tends to yield a better fit for experimental data, mainly when chemical adsorption is the rate‐limiting step. The theoretical basis for the pseudo‐second‐order kinetic model is derived from Langmuir's dual‐area adsorption model, which aligns more closely with the universal rate law governing chemical reactions.^[^ ^48^ ^]^

(4) $$\ln\left(q_{e}-q_{t}\right)={\text{lnq}}_{e}-k_{1}.t$$

(5) $$\frac{t}{q_{t}}=\frac{1}{k_{2}.q_{e}^{2}}+\frac{1}{q_{e}}t$$

In this context, *q*_e_ signifies the amount of substance adsorbed at equilibrium, while *q*_t_ denotes the amount of substance adsorbed at a given time *t*. The pseudo‐first‐ and pseudo‐second‐order rate constants are denoted by the terms *k*_1_ and *k*_2_, respectively, while the elapsed time is denoted by *t*.

**Figure 14:**
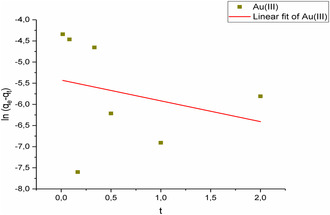
Pseudo‐first‐order modeling of Au(III) ions (pH 2, adsorbent amount 10 mg, Au(III) concentration of1000 μg L^−1^, temperature 298 K).

**Figure 15:**
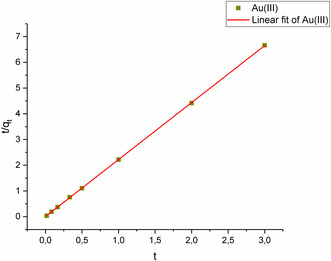
Pseudo‐second‐order modeling of Au(III) ions (pH 2, adsorbent amount 10 mg, Au(III) concentration of1000 μg L^−1^, temperature 298 K).

Solutions containing Au(III) ions were adsorbed from aqueous media over a duration ranging from 1 to 180 min. Both pseudo‐first‐order and pseudo‐second‐order kinetic modeling were employed to interpret the data. **Figure** **14** and **15** illustrate that the pseudo‐second‐order kinetic model (*R* ^2^ > 0.99) is a more suitable fit compared to the pseudo‐first‐order model (*R* ^2^ > 0.55) for the adsorption of Au(III) ions onto Fe_3_O_4_@SiO_2_@CPTES@DAT nanoparticles. The equations and calculated constants corresponding to these kinetic models are detailed in **Table** **4**.

**Table 4:** The parameters of kinetic modeling for Au(III) adsorption (pH 2, adsorbent amount 10 mg, Au(III) concentration of1000 μg L^−1^, temperature 298 K).

Pseudo‐first‐order	q_e_ [mg g]	k_1_ [1/h]	R^2^	Equation
	0.491	0.00441	0.0737	*y* = −0.491–5.42
Pseudo‐second‐order	q_e_ [mg g^−1^]	k_2_ [g h.mg^−1^]	R^2^	Equation
	0.451	26.1	0.999	*y* = 2.21*x* + 0.00324

#### 3.2.8 Analytical Figures of Merit

The RSD, LOD, and LOQ were evaluated as analytical figures of merit and are detailed in **Table** **5**. Eight solutions with 1000 μg L^−1^ of Au(III) ions were adsorbed, and both the mean and standard deviation of the results were calculated. The RSD was determined to be 2.51% by dividing the standard deviation by the mean and multiplying by 100. Additionally, eight blank solutions were analyzed under optimal conditions to ascertain the standard deviation of the results, and the slope of the calibration curve after preconcentration was calculated. The LOD was established at 1.18 μg L^−1^ by multiplying the standard deviation by 3 and dividing by the slope, while the LOQ was calculated to be 3.95 μg L^−1^ by multiplying the standard deviation by 10 and dividing by the slope.

**Table 5:** Analytical figures of merit.

RSD	2.51%
LOD	0.019 μg L^−1^
LOQ	0.065 μg L^−1^

#### 3.2.9 Water Sample Analysis

The concentration of Au(III) ions in drinking water, tap water, wastewater, and stream and river samples taken from different areas of Sakarya, Türkiye, was evaluated using the magnetic solid phase extraction method. The samples underwent adsorption and desorption processes under the optimal conditions established in the study, and the concentration of Au(III) was measured using ICP‐OES. The findings revealed that Au(III) concentrations in all samples were below the detection limit. However, in the spiked samples, the concentrations of Au(III) were successfully recovered with high efficiency. The recovery efficiencies for the increased amounts of Au(III) ions, 500 and 1000 μg L^−1^, ranged from 96.4% to 111.2%, as shown in **Table** **6**.

**Table 6:** Utilization of the proposed methodology for the analysis of water samples (pH 2, adsorbent amount 10 mg, contact time 20 min, desorbing agent 1 mol L^−1^ HCl (0.1 mol L^−1^ thiourea)).

Samples	Spiked concentration [μg L^−1^]	Found concentration [μg L^−1^] ± SD	Recovery [%]
Drinking water	0	ND	–
	500	482 ± 9	96.4 ± 1.8
	1000	1034 ± 21	103.4 ± 2.1
Tap water	0	ND	–
	500	498 ± 5	99.6 ± 1.0
	1000	1015 ± 13	101.5 ± 1.3
Wastewater	0	ND	–
	500	491 ± 3	98.2 ± 0.6
	1000	959 ± 5	95.9 ± 0.5
Stream water	0	ND	–
	500	516 ± 13	103.2 ± 2.6
	1000	1112 ± 18	111.2 ± 1.8
River water	0	ND	–
	500	492 ± 5	98.4 ± 1.0
	1000	975 ± 1	97.5 ± 0.1

ND: not determined

#### 3.2.10 Comparison with Other Methods

The findings of this study were compared to various magnetic solid phase extraction methods reported in the previous research for the adsorption of Au(III) ions, as detailed in **Table** **7**. When assessing performance parameters such as maximum adsorption capacity, LOD, and RSD, the proposed method demonstrated results comparable to those of other methods.

**Table 7:** A comparison between prior magnetic solid phase extraction publications and the applied approach.

Adsorbent	q_m_ [mg g^−1^]	LOD [μg L^−1^]	RSD	Ref
NiFe_2_O_4_	34.6	1.32	1.57	[40]
CoFe_2_O_4_@SiO_2_@[BMIM]PF_6_	–	0.001	3.4	[49]
Fe_3_O_4_@Polythiophene	–	2	3.3	[50]
Fe_3_O_4_@SiO_2_@bis(3‐triethoxysilyl propyl)tetrasulfide	60.2	0.7	1.1	[51]
Fe_3_O_4_@Amidosulfonicacid	–	0.3	6.0	[52]
Fe_3_O_4_@SiO_2_‐SH	84.75	–	–	[53]
Fe_3_O_4_@SiO_2_@CPTES@DAT	11.6	0.019	2.51	This work

## 4 Conclusion

In this study, novel triazole‐coated MNPs were aimed to be synthesized for the recovery of Au(III) ions. Fe_3_O_4_ nanoparticles were coated with SiO_2_, CPTES, and DAT to enhance their oxidation resistance and to introduce functional groups that facilitate the recovery of Au(III) ions. The morphology, size, shape, and coating effectiveness of the nanoparticles were characterized using various techniques, including FESEM, EDX, XRD, FT‐IR, TGA, and DTA. During the optimization studies, we examined several parameters such as pH, adsorbent amount, contact time, and desorption. The findings revealed that the extraction of Au(III) ions was most efficient in a low pH environment with a minimal adsorbent amount and a brief contact time. Furthermore, Au(III) ions were desorbed from the nanoparticle surface using thiourea in an acidic medium. Adsorption isotherm and kinetic modeling were conducted under optimal conditions, demonstrating that the studied method is best described by the Freundlich isotherm and pseudo‐second‐order kinetics. The analytical figures of merit indicated that the method exhibits low limits of detection and relative standard deviation values. To validate the method's applicability for real‐world situations, Au(III) ions were aimed to recover from water samples. The samples were taken from different parts of Sakarya province in Türkiye. The findings demonstrated that the Au(III) levels in the samples examined were below the detection limit. The synthesized Fe_3_O_4_@SiO_2_@CPTES@DAT nanoparticles show significant potential for use in the recovery of Au(III) for a variety of applications.

## Conflict of Interest

The authors declare no conflict of interest

## Author Contributions

**Celal Caner:** investigation (lead) and software (lead). **Miraç Salpat:** data curation (lead) and investigation (lead). **Salma Tabassum:** project administration (equal) and writing—review and editing (equal). **Nuray Canikoglu:** validation (lead) and visualization (lead). **Huseyin Altundag:** project administration (lead); supervision (lead); and writing—original draft (lead).

## Data Availability

The data that support the findings of this study are available on request from the corresponding author. The data are not publicly available due to privacy or ethical restrictions.
